# Catastrophic expenditure due to out-of-pocket health payments and its determinants in Colombian households

**DOI:** 10.1186/s12939-016-0472-z

**Published:** 2016-11-10

**Authors:** Jeannette Liliana Amaya-Lara

**Affiliations:** Instituto de Salud Pública, Pontificia Universidad Javeriana, Bogotá, Colombia

**Keywords:** Expense distribution, Out-of-pocket expenditure, Catastrophic spending, Health insurance policy, Health economy

## Abstract

**Background:**

Out-of-pocket expenditure to pay for health services could result in financial catastrophe. The purpose of this study was to identify the incidence and determinants of catastrophic out-of-pocket payments for healthcare in Colombia. The underlying hypotheses are that low-income and non-insured population in Colombia, and households living in isolated and high level of rurality regions, are more likely to incur catastrophic healthcare expenses.

**Methods:**

This study used data from the Quality of Life National Survey conducted in Colombia in 2011. The presence of catastrophic healthcare spending was calculated using the methodology proposed by the World Health Organization in 2005. Households were classified as having catastrophic health spending when their out-of-pocket health payments were over 20 % of their payment capacity. All other households were classified as not having catastrophic health spending. A probit model was estimated aimed at determining what factors influence the probability of catastrophic healthcare spending.

**Results:**

Study findings show that 9.6 % of Colombian households had catastrophic expenditure. The incidence was higher in households in the Pacífica and Atlántica regions, extended and nuclear families, households with children or elderly adults, located in rural areas, and not insured under the healthcare system. The ratio of household members who work seems to reduce the risk of catastrophic healthcare spending, but the occurrence of any in-patient event increases it. So, there is no statistical evidence for rejecting the hypotheses under study.

**Conclusions:**

Results indicate the importance of establishing intervention mechanisms in order to improve equity in access and payment for health care, protect vulnerable groups against financial risk, and, consequently, reduce the incidence of catastrophic healthcare spending. For this, it is essential to achieve universal health coverage through standardized and improved health services packages for vulnerable age groups and implement healthcare campaigns for households in rural areas where the incidence of out-of-pocket payments is higher.

## Introduction

The Colombian health system has improved the access to healthcare services since the 1993 reform, thanks to the creation of subsidized and contributive healthcare policies. Coverage was extended to the family of the employees insured to contributive policies, and the low-income population was included in the subsidized policies, thus improving access to healthcare services [1]. The health insurance system implemented as of the reform aimed at efficiently achieving equitable quality universal coverage; however, the health system cannot yet efficiently guarantee equitable financial protection of all households, and sometimes households are forced to pay for healthcare services themselves leading to catastrophic spending when they do not have the capacity to pay.

Given that catastrophic spending is calculated as a relative measure of the capacity to pay, it is possible for any household, regardless of its socioeconomic bracket, to incur in catastrophic spending. In this regard, the analysis of healthcare expenses is an important contribution to understanding the financial situation in Colombian households. Likewise, this research looks for identify the groups of the population most vulnerable to catastrophic healthcare spending taking into account their socio-demographic, socioeconomic, and geographic conditions, as a valuable tool for political decision-makers in the challenging quest for universal healthcare coverage and for efficient, equitable Colombian healthcare service provision.

The hypotheses under study are that Colombia low-income population and those who are not insured under the health system do not have the economic capacity to pay for sudden health problems. Likewise, households living in isolated and high level of rurality regions are more likely to incur in catastrophic healthcare expenses. To confirm or reject those hypotheses, data from the Quality of Life National Survey (2008) was analyzed. As studies related to catastrophic expenditures in Colombia are scarce, this research will sheds light on the features that can make households in Colombia more vulnerable to catastrophic expenses, in order to confirm or reject the hypotheses under study.

## Background

1993 Law 100 was created to confront an inequitable healthcare system, with serious access and quality issues for most Colombians. Since the enactment of this Law, all Colombians will participate in the essential health service that allows the General Social Security Health System (SGSSS in the Colombian acronym), some of them will do as members of the subsidized or contributive scheme and others will do as so-called non-insured who are not affiliated to any health insurance.

Members of the system by the subsidized regime are people the poorest and most vulnerable population unable to pay to cover the total amount of the contribution and require total or partial subsidy to be able to receive healthcare services. This population is targeted by the Identification System for Potential Program Beneficiaries (SISBEN is the Colombian acronym) created in 1993. This system enables classifying the population in a scale from 1 to 6 based on its economic wellbeing, which is evaluated from their access to public utilities, possession of durable goods, and allotment of human capital and of current income [2]. Persons placed in levels 1 and 2 in the SISBEN benefit from total or full subsidy under the subsidized social security health policy, provided that such persons are not and should not be insured under the contributive healthcare policy.

The insurance under the contributive healthcare policy is mandatory for people with employment contract, pensioners or retirees and independent workers with capacity to pay. This affiliation also covers the affiliated person’s family and offers benefits such as payments for sick leave and maternity leave, which are not available to the subsidized population [3]. Notwithstanding these benefits, there are independent workers insured under the subsidized health policy, who not feel obliged to enter the contributive policy and would rather stays insured under the subsidized health policy thus evading the system, even though they have the income required to be insured in the contributive social security scheme (﻿more than one ﻿current legal minimum monthly salary) [4, 5].

Non-insured households, that is to say, those where no member is part of the contributive or subsidized scheme﻿s, have a higher risk of incurring in catastrophic expenses due to the fact that the most of the costs of health events must be paid out-of-pocket and such amounts can be very high, especially for low-income households [6, 7]. Further, in spite of the so﻿cial securit﻿y act's﻿ introduct﻿ion in 1993, in the year 2011 there﻿ was still 9.1 % of the Colombian population not insured under any regime in the SGSSS [8], thus facing the possibility of catastrophic expenses due to out-of-pocket healthcare expenditure. However, this situation can also be in households covered by a health insurance policy, and it can be associated with factors such as income level, household configuration, health situation, and health events, among others [9].

Efforts made in recent years have resulted, among others, in increased SGSSS coverage, better access and use of healthcare services, less inequality among income levels and geographic areas, and reduced out-of-pocket healthcare expenditure [10]. However, cases of catastrophic spending are still present in Colombia, and it is a situation that must be counteracted because it affects the most vulnerable groups of the population, that is, households in rural areas and households with the highest levels of poverty [11, 12]. According to the 2007 National Health Survey, 22.1 % of Colombian households lived in rural areas and 49.6 % was placed in SISBEN levels 1 and 2 [13]. This population faces a high financial vulnerability when it has out-of-pocket healthcare expenditure.

Several studies have evaluated catastrophic healthcare spending in different countries; they have found a set of possible factors that may influence the probability that a household incurs in catastrophic expenses due to out-of-pocket healthcare expenditure, even if the level of influence of such factors may vary depending on the level of development of the country under analysis. Among such factors is the existence of health insurance, the type of health event that required for health service payments, the economic situation of the households, socio-demographic conditions, and some characteristics of the head of household.

Among the Latin-American countries, Colombia has the most expensive medicine; this affects the low-income population usually insured under the subsidized health policy or not insured under any health policy [14]. Therefore, health expenses can become catastrophic healthcare spending not only after an event requiring hospitalization (with or without surgery) for a given period of time [15] but also due to payments associated with outpatient events and/or purchase of medicine [16].

Likewise, studies have found that households with a lower income level are more vulnerable to catastrophic healthcare spending [17, 18]. Seeking care in a public or private hospital increases the risk of catastrophic health expenditure [19]. Households in which a member of the family is unable to work and households including members over 60 years of age or children are also more susceptible to catastrophic healthcare spending [20–22].

A research study was conducted in 59 countries worldwide. Colombia ranked fourth among the countries with the highest percentage of households with catastrophic healthcare spending (6.3 %); Azerbaijan ranked third (7.2 %); Brazil, second (10.3 %); and Vietnam, first (10.5 %). Out of the 10 Latin-American countries researched: Brazil, Colombia and Argentina had the highest percentages of catastrophic healthcare spending; the other nine (9) Latin-American countries had percentages no higher than 3.6 % [7] with Mexico having the lowest levels (1.5 %). However, many other studies regarding out-of-pocket healthcare expenditure and catastrophic healthcare spending have been conducted in Mexico [17, 18, 23–26].

Additionally, there have been comparative studies among the different Latin-American countries. Castro (2012) presents a full comparative study of the models, processes and results of the healthcare system in Colombia, Brazil, Mexico, Chile and Costa Rica [14]. Perticara (2008) researched the incidence of out-of-pocket healthcare expenditure in Colombia, Brazil, Mexico, Chile, Argentina, Ecuador and Uruguay; findings showed that catastrophic healthcare spending seems to be associated with high out-of-pocket healthcare expenditure often related to in-patient services rather than with the low payment capacity of the household [27].

Also, Knaul et al. [28] carried out an analysis on the level of catastrophic healthcare spending and its determining factors in 12 Latin-American countries, including Colombia [28]. Results showed, with evident heterogeneity among countries, that out-of-pocket healthcare expenditure is leading a large portion of the population to poverty and that the most vulnerable segments of society are also those with greater risk of a financial catastrophe due to out-of-pocket healthcare expenditure. Household factors, such as being located in rural areas, being in the lowest income quintiles, having elder members in the household, and the lack of health insurance, are associated with a greater probability of incurring in catastrophic healthcare spending.

Alvis et al. [29] developed a study in Cartagena de Indias, Colombia [29]. They found that households in low socio-economic brackets, in which the head of household has no health insurance, little education, and is unemployed or is an independent worker, have a greater probability of having catastrophic healthcare spending. Findings also showed that the method of financing healthcare associated with out-of-pocket healthcare expenditure has become a barrier for household access to healthcare services.

Amaya and Ruiz (2011) conducted a research study in Bogotá, Colombia, consisting of a monthly follow-up of the income and expenses in Bogotá households during a one-year period [9]. Findings showed that 5 % of Bogotá households had catastrophic healthcare spending, in particular, those with low income, without health insurance and with a head of household over 60 years of age. The researchers also found that out-of-pocket healthcare expenditure for outpatient healthcare services and for medicine, rather than out-of-pocket healthcare expenditure from events that required in-patient services had a significant influence in the probability of incurring in catastrophic healthcare spending.

Based on the 2008 Quality of Life National Survey, Gil et al. [30] researched the determining factors of out-of-pocket healthcare expenditure and of catastrophic healthcare spending in the Central region of Colombia, and found that the main factors that influence the probability of catastrophic healthcare spending are: the presence of women of childbearing age in the household, the gender and age of the head of household, and any household members with chronic diseases. However, it is worth mentioning that other variables, such as health insurance, the household being located in an urban or rural area, and the income level, were not statistically significant [30].

The World Health Organization has evaluated different methodologies to estimate the payment capacity and the catastrophic healthcare spending in the households. The assumption in the proposal disseminated in 2005 was that payment capacity was non-subsistence expenditure and that catastrophic healthcare spending was out-of-pocket healthcare expenditure that surpassed 40 % of the household’s payment capacity [31]. The threshold used to define the condition of catastrophic healthcare spending may vary depending on the country under study [17, 18, 25, 32–34]. In Colombia specifically, a threshold of 20 % had been used in some studies on this topic, and this was the limit reference percentage considered for this research.

The analysis of healthcare expenditure behavior and of the effects that it has on the total income of the household is important to determine the level of financial protection in the household [35]. Different studies have been conducted regarding the use of healthcare services and out-of-pocket healthcare expenditure [36–40] and there have been recent advances in the effect that out-of-pocket healthcare expenditure has on the economic wellbeing in the household. This topic is vital, given that out-of-pocket healthcare expenditure can be catastrophic to the household, to the point of plunging it into poverty [6], and it can arise due to basic healthcare service payments, not necessarily due to events that require high-cost healthcare services [16].

Moreover, the geographic location of the household could be an important variable to be analyzed, taking into account the regional inequalities in Colombia regarding income, health condition linked to living conditions, difficult access to healthcare services especially in the rural area, among other issues. Based on the findings of this study, health policy decisions can be focused on regional and geographical needs regarding access to healthcare services and the financial protection, aimed at reducing the population’s out-of-pocket healthcare expenditure and, thus, the level of catastrophic healthcare spending.

## Methods

### Definition of catastrophic healthcare spending

Catastrophic healthcare spending is defined in the literature on the topic as a relative measurement of a household’s payment capacity for a given period of time [7, 41], not solely as expenditure in the event of high cost healthcare services such as in-patient services or services for the treatment of chronic illnesses [23]. The World Health Organization has proposed different methodologies for estimating financial protection, which are different from those for measuring payment capacity and for measuring catastrophic healthcare spending.

This study used the methodology proposed by the World Health Organization in 2005 [31], with the assumption that catastrophic healthcare spending are incurred in when the out-of-pocket healthcare expenditure is equal to or greater than a household’s payment capacity threshold. Given that the above is a relative measure of the payment capacity, some households incur in catastrophic healthcare spending due to out-of-pocket healthcare expenditure from healthcare events, which do not necessarily constitute high-cost out-of-pocket healthcare expenditure but that, nevertheless, surpass the household’s payment capacity.

Although a consensus has not yet been reached regarding the threshold as of which healthcare expenses should be considered catastrophic, the World Health Organization has established the threshold at 40 % for developed countries but affirms that this percentage can change depending on the specific situation of the country for which healthcare expenditure is being measured [31]. Studies conducted in Colombia on the topic have established the threshold at 20 %, and this is the threshold that was used for this study in comparative analyses. Therefore, a household was considered to incur in catastrophic healthcare spending if its out-of-pocket healthcare expenditure was equal to or greater than 20 % of its payment capacity.

The methodology proposed evaluated the percentage of out-of-pocket healthcare expenditure regarding each household’s payment capacity, with the assumption that the payment capacity is a household’s total expenditure minus its subsistence expenditure (if the household’s food expenditure is equal to or greater than its subsistence expenditures) or a household’s food expenditure (if the household’s food expenditure is lesser than its subsistence expenditure). A dummy variable for catastrophic healthcare spending is then obtained, which has a value of 1 if the quotient between the household’s out-of-pocket healthcare expenditure and its payment capacity is higher than 20 %; if not, it has a value of 0.

To calculate the above, Xu et al. [7] considered the following definitions: 1.- A household’s subsistence expenditure is the product of the poverty line per capita line and the adjusted household size; 2.- the household size being analyzed is the size of the household weighted by a factor that indicates that consumption increases with additional household members although such increase is less than proportionate to the increase in the size of the household; 3.- the poverty line is the average of the food expenditure values of households whose food share was in the 45th to 55th percentile range of the household’s income; and 4.- the equivalent food expenditure is the quotient of the household’s food expenditure and the adjusted household size.

### Data

This study used the information obtained from the 2011 Quality of Life National Survey that gathers data on Colombian household expenditure during the last month considering different items, including healthcare service payments. The survey was representative for the municipal hubs and the rural areas of the following large Colombian regions (Antioquia, Valle, Atlántico, Pacífica, Central, Oriental), plus just the urban areas of the Orinoquía-Amazonía region, as well as Bogotá and San Andrés departments. In 2011 it included the Provincial Departments of Guajira, Córdoba, Boyacá, Cauca, Chocó and Nariño [42].

The study universe comprised the non-institutional resident civilian population throughout Colombia. Although information on individuals was available, aggregate household data was used for this research, given that the socio-demographic conditions are the same for all the members of a household and, in general, income and healthcare expenses tend to be shared within a family group.

The survey was given to 25,364 households; the catastrophic healthcare spending variable could not be calculated for 123 of those households because they did not report expenses for the month under study or because their payment capacity was null because they reported the exact same amount in total household expenses as in food expenses.

The reference period varied for different types of expenses; some were measured weekly whereas others were measured monthly, quarterly or annually. Therefore, a standardization process was necessary for the reference periods being analyzed, to give the same time unit to all of the expenses: monthly values. In this fashion, it was ultimately possible to obtain a household’s monthly expenses, which enabled calculating the catastrophic healthcare spending variable.

### Defining the variables

Aimed at identifying the factors that lead households to incur in catastrophic healthcare spending, a dichotomous variable was used as a dependent variable with the value 1 if the household had catastrophic healthcare spending or with the value 0 if it did not. Possible explanatory variables are geographic characteristics, household configuration, health variables, and socioeconomic condition. Due to the underreporting seen in the income variable, the income proxy used was each household’s total expenditure. The definition for each one of the variables is shown in Table 1.

**Table 1:** Possible explanatory factors for catastrophic healthcare spending

Explanatory Variables	Definition
Region Pacífica (not including Valle) Atlántica Oriental Central Valle Orinoquía-Amazonía Antioquia Bogotá San Andrés	Region of Colombia in which the household is located
Area Urban Rural	Residential area of the household
Household Size	Number of persons living in the household during the reference month
Type of Family One person Nuclear Extended Composite	Type of family by category based on its configuration: Nuclear: a couple with or without children or a single parent with children Extended: a couple or a single parent, with or without children, and other relatives Composite: families with persons who are not relatives
Vulnerable Household Members Children and elderly adults No children but elderly adults Children but no elderly adults No children and no elderly adults	Vulnerable household members means children five years old or younger and elderly adults 65 years old or older
Head of Household’s Age Younger than 65 years old 65 years old or older	Dummy variable for identifying heads of household (hh) 65 years old or older
Head of Household’s Gender Male Female	Dummy variable for identifying female heads of household (hh)
Perception of Heath Condition Bad or poor Good Different perceptions	Each household member’s perception of his or her health condition. Households where some members consider themselves in good health and others in bad or poor health are classified as “Different perceptions”.
Health Insurance Policy Contributive Subsidized Special Non-insured Combined	Each household member’s type of affiliation to the SGSSS during the reference month. “Combined” indicates that not all household members have the same type of affiliation.
Type of Healthcare Service Paid Outpatient Outpatient and medicine Medicine Any in-patient event None	Type of healthcare services for which the household incurred in out-of-pocket healthcare expenditure. In particular, outpatient healthcare services included doctor’s appointments, dentist’s appointments, vaccination, blood tests, Rx and alternative therapy.
Income Quintile Quintile I Quintile II Quintile III Quintile IV Quintile V	Household income quintile based on the average total expenditure during the reference month. Quintile I groups 20 % of the households with the lowest income, whereas Quintile V groups 20 % of the households with the highest income.
Ratio of Household Members Who Work	Number of household members who work out of the total number of household members

Even though the studies on catastrophic healthcare spending consider the characteristics of the head of household as explanatory variables, household configuration is very peculiar in Colombia because many households comprise not only a couple and their children but also close family members, distant family members and even persons who are not related to the family. Such configuration generates inequality in the characteristics of the household associated with health insurance, work situation, and health conditions. However, income tends to be distributed according to the needs of all the members of the household and, therefore, healthcare expenses affect the entire household, not just one member of the household in particular. For this reason, type of family [43] was included as an explanatory variable (see Table 1).

### Multivariate model

Aimed at determining the factors that have some influence on the presence of catastrophic healthcare spending and its magnitude, the application of a binary response model was proposed. This model uses a dummy variable as a dependent variable, which indicates if the household had (1) or did not have (0) catastrophic healthcare spending during the month under study.

This variable was used with factors such as region, area, household’s configuration, number of children or adults, health insurance policy, health perception, income, among other variables. After all the independent variables were analyzed for correlation and association, those that showed significance on a bivariate level and had theoretical relevance were selected.

The probit binary response model used variables restricted to a value from zero (0) to one (1) for all real numbers, which ensured that the estimated probabilities were within the same range [44] and enabled establishing the magnitude and the intensity of the relation between each independent variable and the household’s catastrophic healthcare expense condition.

The probit model was defined by:

$$ P\left(y=1/x\right)=G\left({\beta}_0+{\beta}_1{x}_1+\dots +{\beta}_k{x}_k\right)=G\left({\beta}_0+x\beta \right) $$

where G is the accumulated distribution function of the standard normal, *β*_0_ is the model constant, *x* corresponds to the set of independent variables and *β* is the parameter vector.

## Results

Descriptive statistics of explanatory factors for catastrophic healthcare spending and probit model to identify the probability of incurring in catastrophic healthcare spending are analyzed. Stata 13 was used for all analysis [45].

### Catastrophic expenditure by regime and geographical distribution

**Fig. 1:**
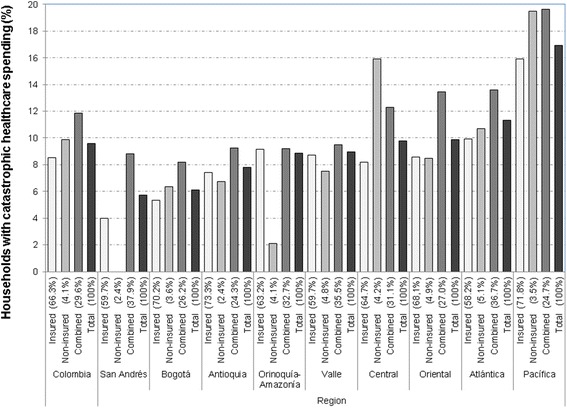
Percentage of Households with Catastrophic Healthcare Spending, according to region and health insurance policy

The analysis of possible explanatory factors for catastrophic healthcare spending was conducted, considering the percentage of households with catastrophic healthcare spending and the 95 % confidence interval (95 % CI) estimated. Figure 1 shows the percentage of households with catastrophic healthcare spending, by region and by household’s insurance status. The X-axis shows the percentage of households by insurance status in each region, in order to know the population from which the percentage of households with catastrophic healthcare spending was calculated.

The results shows that 9.6 % of Colombian households incurred in catastrophic healthcare spending, with differences according to the region: households in the Pacífica region were the most vulnerable to incurring in catastrophic healthcare spending (16.9 %; 95 % CI = 15.9 %-18.1 %), followed by Atlántica (11.3 %; 95 % CI = 10.2 %-12.5 %), Oriental (9.9 %; 95 % CI = 8.7 %-11.3 %) and Central (9.8 %; 95 % CI = 8.4 %-11.4 %) regions, whereas only 6.1 % of households in the Bogotá (95 % CI = 4.8 %-7.7 %) and 5.7 % of households in San Andrés (95 % CI = 4.0 %-8.1 %) had catastrophic healthcare spending.

These percentages differ depending on the household’s insurance condition. In Colombia, 8.5 % of the 66.3 % of the households with members in the same type of affiliation during the reference month incurred in catastrophic healthcare spending and it increases to 9.9 % in non-insured households (4.1 %) and to 11.8 % in households with members in different affiliation condition (29.6 %). In San Andrés, 59.7 % of households are insured, 2.4 % are non-insured and 37.9 % have members with different type of affiliation. None of non-insured households had catastrophic spending, but 8.8 % of households with combined affiliation and 4.0 % of insured households had problems of financial protection against healthcare expenses.

Bogotá and Pacífica regions have similar distribution of households by health insurance policy, but with differences in the percentages of catastrophic healthcare spending. In Bogotá, 5.3 % of insured households (95 % IC = 3.9 %-7.2 %), 6.4 % of the non-insured households (95 % IC = 5.1 %-8.0) and 8.2 % of households with combined affiliation status (95 % IC = 5.6 %-11.8 %) had catastrophic healthcare spending. These percentages respectively increase to 15.9 % (95 % IC = 14.7 %-17.2 %), 19.5 % (95 % IC = 18.3 %-20.8 %) and 19.6 % (95 % IC = 17.4 %-22.1 %) in Pacífica region.

A similar situation occurs in Valle and Atlántica regions, where the distribution by type of household health insurance policy is similar, but the percentage of households with catastrophic expenditure within each group differs. In Valle, the percentage of catastrophic healthcare spending is higher in households with combined affiliation (9.5 %; 95 % IC = 7.3 %-12.3 %), followed by insured (8.7 %; 95 % IC = 6.7 %-11.4 %) and non-insured (7.5 %; 95 % IC = 4.3 %-12.9 %) households. In contrast, the occurrence of catastrophic healthcare spending in Atlántica region is 13.6 % (95 % IC = 11.6 %-15.9 %), 9.9 % (95 % IC = 8.6 %-11.4 %) and 10.7 % (95 % IC = 9.5 %-11.9 %), respectively.

On the other hand, distribution by health insurance policy in Orinoquía-Amazonía and Central regions is quite similar: slightly over 63 % are insured households, close to 4 % are non-insured households and about 31 % are households with combined affiliation. However, in the Orinoquía-Amazonía region the occurrence of catastrophic healthcare spending is higher in households with combined affiliation (9.2 %; 95 % IC = 5.6 %-14.8 %) and insured households (9.1 %; 95 % IC = 6.7 %-12.3 %) than in non-insured households, but in Central region the highest percentage was found in non-insured households (15.9 %).

Results show that in Colombia, and specifically in Central, Atlántica and Pacífica regions, the percentage of catastrophic expenditure is higher among insured households than among non-insured households, which may be due to lower healthcare seeking by the non-insured population, possibly because of geographic barriers or economic factors. The percentage of catastrophic healthcare spending in Oriental region is similar among insured and non-insured households. These results indicate the need for greater financial protection for households living in these regions of the country, in order to reduce the out-of-pocket healthcare payments.

The differences on the occurrence of catastrophic healthcare expenditure among regions could be due to the urban–rural distribution. In Colombia, the distribution of health insurance policy differs by areas: there are more households insured under the subsidized health policy (57.1 %) and non-insured (5.8 %) in the rural areas, whereas in urban areas the affiliation to contributive and special regimes is higher (40.7 % and 2.2 % respectively). The percentage of catastrophic healthcare spending in non-insured households was higher than in insured households into both areas: 7.4 % versus 6.9 % in the urban areas and 14.1 % versus 15.5 % in the rural areas of the country. Likewise, the percentage of households with catastrophic healthcare spending is higher in rural areas than in urban areas in all regions and it tends to increase in regions with greater rurality.

### Determinants of catastrophic expenditure

A binary response model was estimated to determine the factors with a statistically significant impact on the incurrence in catastrophic healthcare spending and its magnitude. The model used a dummy variable as a dependent variable, which indicated if a household had (1) or did not have (0) catastrophic healthcare spending during the reference month. The process carried out to specify the model ensures the absence of co-linearity and the appropriate adjustment of the model.

Starting with the possible explanatory factors for the incurrence in catastrophic healthcare spending, it was decided to exclude the residence area from the model because most low-income households are located in rural areas and, therefore, there is a high degree of association between those two variables (pearson chi2(4) = 3.4e + 03; *p*-value < 0.001). Work in the informal sector and perception of health condition were also excluded from the model due to their association with health insurance affiliation (*p*-value < 0.001). Furthermore, the head of household’s gender was not significant at a bivariate level, and the head of household’s age was redundant, considering explanatory variable of presence of vulnerable persons in the household (<=5 years old and/or > =65 years old).

Table 2 shows the probit model results, specifying the coefficients, their standard error, the z-statistic, associated *p*-values, the 95 % confidence interval of the coefficient, and the statistical tests that evidence the confidence level of the results. The Hosmer-Lemeshow test accepts the goodness-of-fit hypothesis (*p*-value = 0.306), the model makes the correct classifications 87.32 % of the time, the area under the ROC curve indicates that this model is acceptably similar to the perfect model (0.666) and the null hypothesis of good model specification is accepted (*p*-value = 0.953).

**Table 2:** Probit model: Probability of incurring in catastrophic healthcare spending

Variables		Coef.	Std. Err.	z	P > |z|	[95 % CI]	
Region^a^	Atlántica	−0.229	0.041	−5.610	0.000	−0.310	−0.149
	Oriental	−0.274	0.047	−5.790	0.000	−0.366	−0.181
	Central	−0.295	0.053	−5.560	0.000	−0.399	−0.191
	Valle	−0.297	0.060	−4.920	0.000	−0.415	−0.179
	Orinoquía-Amazonía	−0.379	0.078	−4.830	0.000	−0.532	−0.225
	Antioquia	−0.412	0.062	−6.610	0.000	−0.534	−0.290
	Bogotá	−0.413	0.072	−5.740	0.000	−0.554	−0.272
	San Andrés	−0.465	0.095	−4.890	0.000	−0.651	−0.278
Type of Family^b^	Nuclear	−0.071	0.041	−1.740	0.082	−0.151	0.009
	One person	−0.011	0.075	−0.140	0.887	−0.158	0.136
	Composite	−0.481	0.188	−2.560	0.010	−0.849	−0.113
Household Members < =5 and/or > =65 years old		0.116	0.038	3.040	0.002	0.041	0.192
Health Insurance Policy^c^	Subsidized	0.306	0.055	5.580	0.000	0.198	0.413
	Combined	0.296	0.056	5.310	0.000	0.187	0.405
	Non-insured	0.257	0.087	2.940	0.003	0.086	0.427
	Special	−0.015	0.174	−0.080	0.933	−0.357	0.327
Any In-patient Event		0.767	0.052	14.760	0.000	0.665	0.868
Income Quintiles^d^	Quintile IV	0.097	0.070	1.390	0.166	−0.040	0.234
	Quintile III	0.203	0.067	3.040	0.002	0.072	0.334
	Quintile II	0.336	0.069	4.850	0.000	0.200	0.471
	Quintile I	0.211	0.071	2.980	0.003	0.072	0.350
Ratio of Members Who Work		−0.344	0.068	−5.060	0.000	−0.477	−0.210
Constant		−1.373	0.088	−15.580	0.000	−1.545	−1.200

Reference categories: ^a^Pacífica, ^b^Extended, ^c^Contributive, ^d^Quintile V

Hosmer-Lemeshow (p-value) 0.306

Cases correctly classified (%) 87.32

Area under ROC curve 0.666

Linktest prediction value p > |Z| 0.000

Prediction squared value p > |Z| 0.953

The model results showed that the region in which households are located significantly influences the probability of incurring in catastrophic healthcare spending. As compared to the households in the Pacífica region, the households in any other region have a lower probability of catastrophic spending, which is coherent with the descriptive analysis finding that the Pacífica region had the highest level of catastrophic healthcare spending (16.9 %). Households in Bogotá and San Andrés, without rural area, have less probability to face catastrophic spending due to healthcare payments. This finding confirms one of the hypotheses under study.

As compared to extended families, nuclear families have less probability of incurring in catastrophic healthcare spending, which can be explained by the increased number of family members, generally associated with elderly persons with a greater risk of health issues. Composite families also have less probability of incurring in catastrophic healthcare spending possibly due to the fact that the members who are not part of the family are not necessarily in vulnerable age groups.

Children and the elderly have a greater risk of health issues and require more medical care than the rest of the population, which influences the probability of their household incurring in catastrophic healthcare spending. In particular, households with members five years old or younger and/or members 65 years old or older have higher probability of incurring in catastrophic healthcare spending than households that have no members in those two age groups.

Health insurance affiliation also determines the incurrence in catastrophic healthcare spending. As compared to households insured under the contributive health policy, households where the members are insured under the subsidized health policy and households without any health insurance have higher probability of incurring in catastrophic healthcare spending, which evidences lower financial protection for the population insured under these two health policies, as it was posed in the hypotheses of this study. As to the type of healthcare services, households in which events requiring in-patient services occurred had more probability of catastrophic spending.

Regarding socio-economic characteristics, each member of the household who works reduces the probability that it will incur in catastrophic healthcare spending, situation that is evidently associated with the higher payment capacity of those households. Likewise, the relation between the household’s income level and its payment capacity shows that the lower the household’s income quintile, the higher the probability of incurring in catastrophic healthcare spending, which corroborates that low-income households are more likely to incur in catastrophic healthcare expenses.

## Discussion

Substantial modifications have been made to the social health insurance scheme in Colombia from 1993 Law 100, thanks to the creation of health insurance that enabled an increased level of health insurance coverage in the households. Specifically, the contributive health policy allowed insuring the families of the employees and the subsidized health policy permitted low-income population with no payment capacity to become affiliated, to cover healthcare expenditure. In the past few years, a high level of health insurance coverage has been achieved but difficult focalization, an increase in informal sector jobs, and a lack of incentives have kept a group of the population out of the health system.

Different population surveys are given in Colombia, including the Quality of Life National Survey that enables analyzing a household’s living conditions and the population’s wellbeing. The survey conducted in 2011 was used because, unlike the same survey in later years, it collected information on health expenditure. These data enabled finding evident financial protection issues in Colombian households: approximately 10 % of the households incurred in catastrophic healthcare spending due to out-of-pocket healthcare expenditure. That percentage represented more than one million households in 2011, which is concerning considering that one of the main goals of health insurance is to reduce a household’s out-of-pocket healthcare expenditure and protect them against exaggerated healthcare expenses.

Catastrophic healthcare spending is a topic of vital importance in the Colombian health sector because this factor is directly related to a household’s financial protection. There is still a gap in universal coverage and economic inequity and unequal access to health services for some groups of the population. This research furnishes valuable information regarding the magnitude of this problem in the different regions of Colombia and also the characteristics of the households that face it.

Findings revealed that health insurance affiliation has a significant influence on the probability of a household having catastrophic healthcare spending, As health insurance affiliation is solely mandatory for persons in the formal job sector, persons in the informal job sector ultimately decide whether they want to be insured under the health system or not, and that decision is generally linked to their need for healthcare services more than to their financial capacity to become insured under the contributive policy.

Therefore, there are persons who do have such financial capacity but who choose not to become affiliated because they consider themselves to be in good health; they do not take into account the fact that health issues usually come without warning and could lead to catastrophic healthcare spending. So, it is necessary the State finds mechanisms to attain universal affiliation under the health system for the population. Given the difficulty in targeting that population, standardizing and improving healthcare service packages might be a guarantee for the non-insured population to become affiliated under the healthcare system.

In terms of geographical location, research found a more evident financial protection problem in the Pacífica and Atlántica regions, possibly because these regions have more households living in urban areas, and located in the lower income quintiles, so it would be convenient to consider intervention mechanisms such as promoting campaigns to increase health care at a low cost in rural areas.

This does not mean leaving aside the other regions of the country which also have many households that incur in catastrophic healthcare spending, mainly in rural zones. Between 12 % and 15 % of households in the rural areas of Oriental, Central, Valle and Antioquia regions have to face catastrophic expenses for healthcare services, probably due to their lower income is not enough to cover the direct or indirect payments for health care.

Also, the presence of vulnerable members within a household is a determinant factor to face financial catastrophes because of out-of-pocket healthcare expenses. Households with children five years old or younger and elderly adults 65 years old or older have higher probability of catastrophic healthcare spending, and it would be relevant to focus efforts in improving healthcare services and financial protection offers for these age groups.

Moreover, the in-patient events have a considerable effect on a household’s financial stability and it should be considered in the financial protection equations and benefit packages worked out by the insurance scheme. However, it is also necessary to direct financial protection on expenses associated with medicine and outpatient healthcare services, because some households that solely had those expenses also incurred in catastrophic healthcare spending.

Unlike some studies conducted for specific areas in Colombia, for example for the Central region (Gil et al., [30]) or for Bogotá (Amaya and Ruiz, [9]), this research enables comparing the presence of catastrophic healthcare spending in the different regions of the country, providing a broader view of the situation regarding a household financial protection. The results can be compared to those presented in other studies using the same methodology proposed by the World Health Organization (Xu, [31]); however, it is important to bear in mind temporality of the information that was analyzed.

This research has limitations related to the period of time under study, because the Quality of Life National Survey is a cross-sectional survey that asks for demographic and economic characteristics at the time of the interview and remembrance of use and expenses in different periods of time (week, month, quarter or year) which were taken to a monthly unit for the analysis. The disposition of data could lead to information bias that influence the results obtained; however, unfortunately there are no panel data that enable a more accurate calculation of the level of catastrophic healthcare spending in the country.

It would, therefore, be convenient to consider conducting studies on income and expenditure with longer periods of time. Panel studies would allow identify the economic compensations in the households in terms of borrowing and selling assets when they face with out-of-pocket healthcare expenditures.

## Conclusions

Out-of-pocket healthcare spending is a worrying issue in Colombia because there are still population groups that do not have enough capacity to pay to cover their health expenses, and such expenses could become catastrophic. It is necessary to establish intervention mechanisms in order to improve equity in access to health services and payment for health care, protect vulnerable population against financial risk, and reduce the incidence of catastrophic healthcare spending. The most vulnerable groups are households in the Pacífica and Atlántica regions, extended and nuclear families, households with children or elderly adults, located in rural areas, and not insured under the health system. To reduce the catastrophic healthcare expenditures will be required to seek universal health coverage through standardized and improved health services packages, ensuring financial protection against health-related risks and equity in the health system. Implementation of healthcare campaigns for population in rural zones is also essential to reduce the probability of catastrophic health expenditure in Colombian households.

## Abbreviations

SGSSS: General social security health system
SISBEN: Identification system for potential program beneficiaries
